# Patent foramen oval: A rare case of acute ischemia of the upper limb: A case with 2 year follow-up

**DOI:** 10.1016/j.amsu.2021.102188

**Published:** 2021-02-23

**Authors:** O. Kallel, D. Charmake, I. Chergui, N. El Ouafi, N. Ismaili

**Affiliations:** a Department of Cardiology, Mohammed VI University Hospital of Oujda, Mohammed First University of Oujda, Morocco; b Laboratory of Epidemiology, Clinical Research and Public Health, Faculty of Medicine and Pharmacy, Mohammed the First University of Oujda, Morocco

**Keywords:** case report, Patent foramen oval, Upper limb ischemia, Pulmonary embolism, Paradoxical embolism

## Abstract

**Introduction and importance:**

Paradoxical emboli (PDE) represent less than 2% of all arterial emboli, that is why they are considered as a rare event. We notice that the upper limb ischemia is very exceptional as part of a paradoxical embolism. This case presentation can help in considering the diagnosis the PFO as one of the most important risk factors of paradoxical embolism.

**Case presentation:**

Here, we present a rare case of a 69-year-old woman with paradoxical systemic arterial embolism, presented by an acute ischemia of the upper limb, secondary to deep venous thrombosis and pulmonary embolism in the presence of patent foramen ovale, treated with long-term anticoagulation with rivaroxiban 20 mg/day, because of the mutation of the Factor II whish indicate already the anticoagulation.

**Discussion:**

Echocardiographic techniques such as transthoracic echocardiography (TTE), transesophageal echocardiography (TEE), or transcranial echocardiography (TCE) are the principal tools used to detect PFO. There are no clear consensus on the treatment of PDE. Presenting symptoms largely depend upon the location of the embolus, necessitating a different approach for each patient, but There is essentially three therapeutic options: surgical embolectomy, thrombolysis, and anticoagulation.

**Conclusion:**

PFO closure is, today, a standardized and safe intervention, but the indication stay individualized to each patient.

## Highlights

• Paradoxical emboli represent less than 2% of all arterial emboli, that is why they are considered as a rare event.
• The patent foramen oval stay the most frequent intracardiac defect associated with paradoxical emboli.
• transesophageal echocardiography TEE is the most reliable diagnostic test of the patent foramen oval.
• Recommendations for treatment should be individualized.

## 1 Introduction

The Acute ischemia of the upper extremity is considered as exceptional; only one fifth of the acute limb ischemia is presented in the upper extremity [1]. The pathophysiology and the cause of the upper extremity ischemia depend of the size of the vessel. large vessel artery diseases include essentially atherosclerosis, but more rarely embolic occlusion can be the cause. This embolic occlusion is habitually caused by an atrial fibrillation, but it can also be very exceptionally as part of a paradoxical embolism wish is the case of our patient.

The Paradoxical emboli represent less than 2% of all arterial emboli [2]. It's defined by the passage of venous embolic material into the arterial circulation, passing through an intracardiac right to left shunt. It's described for the first time by Cohnheim in 1877 [3]. The most common intra-cardiac defect, associated with paradoxical emboli, is the patent foramen oval, it's estimated to be present in 25% of the population [4].

This article has been reported in line with the SCARE criteria [5]. It presents an uncommon case of an upper limb ischemia due to a paradoxical emboli associated with a patent foramen oval (PFO).

This case presentation can help in considering the diagnosis the PFO as one of the most important risk factors of paradoxical embolism.

## 2 Case presentation

A 69 year-old female, having arterial hypertension since 5 years as medical history, with a sedentary lifestyle, obesity (BMI: 30.5 kg/m2) and abdominal obesity, with no drug nor family history. She was admitted with symptoms of sudden pain and paresthesia of the left upper limb, started 6 hours before her consultation. During the previous 20 days, she had a progressive dyspnea (class III of the New York Heart Association's classification) with productive cough, causing important limitation of physical activity, for what only symptomatic treatment was taken; this dyspnea had quickly evolved to a class IV of the NYHA's classification, during the last 3 days before her admission. Her family history was negative for any thromboembolic disorders and she denied any history of taking contraceptive drugs.

The physical examination find polypnoea with 24 cycle/min of respiratory rate, and a SatO2 = 91%, blood pressure at 130/90 mm Hg, heart rate at 115 beats per minute (bpm). Cardiopulmonary examination was unremarkable, and extremity examination did not demonstrate any edema, however, a lack of pulse and sensibility with pallor on a cold left upper limb was noticed. The rest of the examination was unremarkable.

The electrocardiogram showed sinus rhythm at 115 bpm, with a right bundle branch block and a negative T wave in V1,V2.

Since the findings on examination of the left upper limb were suggestive of arterial ischemia, an upper limbs angio-CT was performed. The primary interpretations showed a significant arterial thrombosis of the left humeral artery extended to the radial and ulnar arteries. The patient was directly taken to the operating room, where a trans-humeral embolectomy was performed successfully by a junior resident with 4 years of specialised training (using local anesthesia). The intervention adherence and tolerability were well during the whole course of his treatment. Moreover, no other interventions were performed.

**Fig. 1:**
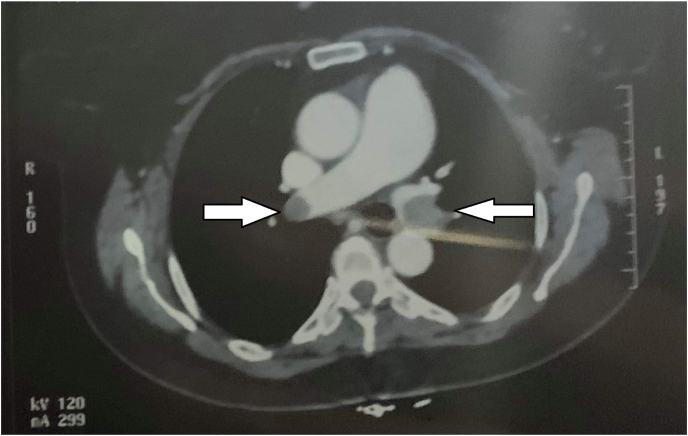
Thoracic computed tomography before any treatment. the two main pulmonary artery are enlarged.

Otherwise, the final interpretation of the angio-CT showed in addition to the arterial thrombosis of the left humeral artery, a massive bilateral Pulmonary Embolism (PE) with a thrombus straddling the two main branches (see Fig. 1).

Lower limb compression ultrasonography showed deep venous thrombosis (DVT) in the right popliteal artery.

Clinical and radiological data gave the suspected diagnosis of paradoxical embolism through a PFO, so a transthoracic echocardiography was performed.

The transthoracic echocardiography showed a dilated, non-hypertrophied right ventricle with a mild systolic dysfunction, an Interatrial septal aneurysm (of 10 mm) and a tricuspid regurgitation allowing calculating the systolic pulmonary pressure: SPP: 67 + 5 mmHg.

**Fig. 2:**
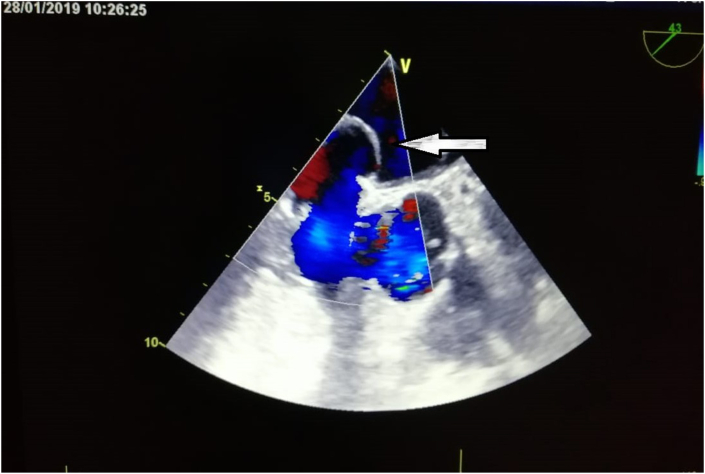
Transoesophageal echocardiography demonstrated an ASIA.

**Fig. 3:**
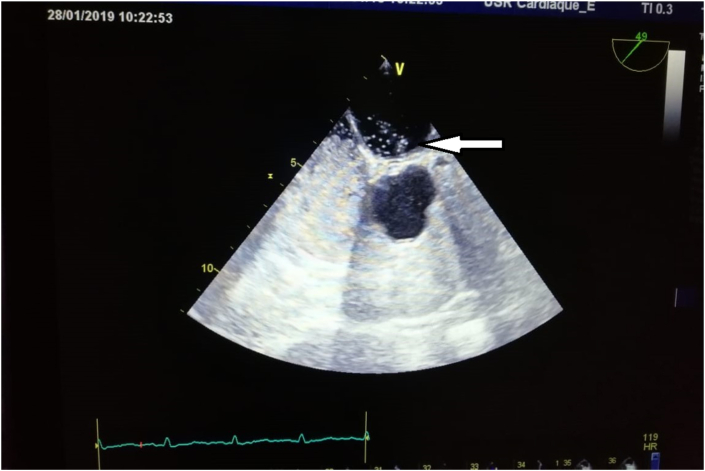
Transoesophageal echocardiography demonstrated positive bubble test.

The TTE did not detect the PFO, so we completed by a trans-esophageal echocardiography (TEE) with a bubble test, which showed an Interatrial septal aneurysm and, at an angle for visualization around 50°, a PFO with a spontaneous positive bubble test (without the Valsalva maneuver) (see Fig. 2, Fig. 3).

The diagnosis of right popliteal DVT and massive PE was proved, with paradoxical embolism in the right upper limb due to a PFO. A medical treatment with enoxaparin 1 mg/kg every 12h was early established, relayed then with direct oral anticoagulants (DOA):Rivaroxiban 20mg/Day.

There were no complications during the hospitalization with a clear clinical improvement. One week after discharge, Transthoracic echocardiography showed improvement of the right ventricular function and the SPP decreased to 40 + 5 mmHg. The electrocardiogram showed a regression of the right bundle branch block.

The patient underwent a thrombophilia screening: Factor V Leiden, antithrombin III, Protein C and Protein S, anticardiolipin immunoglobulin G/immunoglobulin M, and anti-dsDNA antibodies were all negative, but it showed a mutation of the Factor II.

A lifetime anticoagulation with Rivaroxiban was indicated.

After 2 years of follow-up, the patient had no more thrombotic events, with the persistence of PFO with a spontaneous positive bubble test at the TEE and a SPP at 29 + 3 mmHg. No hemorrhagic complications have been noticed during this two years.

## 3 Discussion

The Acute ischemia of the upper extremity is relatively exceptional, only one fifth of the acute limb ischemia is presented in the upper extremity [1]. One of the most uncommon causes of an upper limb ischemia is the paradoxical embolism associated with a patent foramen oval. Arterial embolization is caused only in less than 2% of cases by a paradoxical embolism [2].

Indeed, during the intra-uterine life, the foramen, wish is an interatrial communication, allow oxygenated blood to pass from the right atrium to the left atrium, then to the fetal circulatory system. at birth, as the pulmonary respiration begin, the pressure in the pulmonary vascular and the right atrium abruptly decreases, in the same time, the brutal increase of the venous return beget a greater pressure in the left atrium, hence a functional closure of the foramen oval. It is normally complete before the age of 2 years. When this closing phenomenon does not happen, the foramen ovale persist permeable intro adulthood, it's referred to as a patent foramen oval, which may cause then pathologic right-to-left shunting in some situations when right atrial pressure increases [6].

In the case of PE, right ventricular pressure increases, causing then, if there's a PFO, an arterial right–left shunt, then a paradoxical embolism may be occurred whish was the case of our patient, since the thoracic angio-CT confirmed a PE, and since we noticed a tricuspid regurgitation in the TTE. Otherwise, M. Abad-Arranz and al [7]. reported a paradoxical embolism cases with no tricuspid regurgitation, supposing then that a normal right ventricular pressure would not prevent a thrombus from passing through the PFO.

Echocardiographic techniques such as transthoracic echocardiography (TTE), transesophageal echocardiography (TEE), or transcranial echocardiography (TCE) are the principal tools used to detect PFO [6]. daniels and Al [8]. in a 256 patients' study showed that the TTE have 91% of sensitivity and 97% of specificity. As color Doppler detects only 5%–10% of inter-atrial shunts [9] patients with suspected PFO should undergo a bubble test. The principal limitation of TTE is its relatively poor sensitivity compared to the TEE [6], that's why a TEE with color Doppler should be realized if the transthoracic study is negative or dubious but we still have a strong clinical suspicion of PFO remains [6]. We notice that TEE is the most reliable diagnostic test [10].

In our case, since the TTE did not detect the PFO, we proceeded to a TEE with a bubble test to confirm the PFO. In the bubble study, The presence of a single micro-bubble in the atrium and left ventricle in the first three beats after right cavity opacification confirm the diagnostic of PFO [11], in this case we noticed the appearance of more than 20 bubbles on the left side of the heart in the first three beats after the injection of sterile saline into a peripheral vein without the Valsalva maneuver, knowing that maneuvers that increase right atrial pressure like Valsalva maneuver or coughing improve diagnostic sensitivity [6].

There is no clear consensus on the treatment of PDE. Presenting symptoms largely depend upon the location of the embolus, necessitating a different approach for each patient, but There is essentially three therapeutic options: surgical embolectomy, thrombolysis, and anticoagulation. Recent reviews affirm that surgical thromboembolectomy can reduced significantly the systemic embolism, compared with thrombolytic treatment; added to that more complications occurred with anticoagulation and thrombolysis than enbolectomy [12].

Thrombolysis is more frequently chosen in the higher risk group and is associated with the highest mortality [13,14]. However, the optimal approach will depend on patient characteristics and available hospital resources and expertise [13].

In the present case, transhumeral embolectomy was performed before even the diagnostic of the PE, and the patient had no significant hemodynamic or right ventricular dysfunction (a mild systolic dysfunction) so, After consideration of all options, the patient received only anticoagulation therapy after the transhumeral embolectomy, (A medical treatment with enoxaparin 1 mg/kg every 12 h was early established, then relayed with direct oral anticoagulant: rivaroxaban).

Furthermore, to avoid the recurrence of PDE, PFO closure or antithrombotic medication should be considered. In our case, a PFO closure was not chosen and only the long-term anticoagulation with rivaroxiban 20mg/day was considered, because of the mutation of the Factor II wish indicates already the anticoagulation.

The patient was seen in the consultation of our university hospital one month after the discharge, then every 3 months, and during this 2 years of follow-up, the patient had no more thrombotic events, with the persistence of PFO with a spontaneous positive bubble test at the TEE realized one year after the event.

No hemorrhagic complications have been noticed during this period.

Our patient was satisfied with the quality of our management.

## 4 Conclusion

It is necessary to think about PFO closure in patients with PE and paradoxical embolism, in particular when right heart pressures still elevated. After years of discussions, PFO closure is, today, a standardized and safe intervention in some dedicated catheterization laboratories, even if it's indications still controversial and remain a source of debate because of the lack of data regarding their long-term effects, that's why individualized solutions have to be sought.

## Sources of funding

None.

## Ethical approval

The ethical committee approval was not required give the article type (case report). but we notice that the written consent to publish the clinical data of the patients was given and is available to check by the handling editor if needed.

## Patient consent

Written informed consent was obtained from the patient for publication of this case report and accompanying images. A copy of the written consent is available for review by the Editor-in-Chief of this journal on request.

## Author contribution

- Kallel Oussama: Study concept, Data collection, Data analysis, Writing the paper.
- Darar Charmake: Data analysis.
- Chargui Ismail: Data collection.
- El Ouafi Nouha: data validation.
- Ismaili Nabila: Supervision, study concept, data analysis and data validation.

## Research registration

This is a medical case report not an original research project. This registration is not required.

## Guarantor

Kallel Oussama.

## Provenance and peer review

Not commissioned, externally peer-reviewed.

## Declaration of competing Interest

There are no conflicts.
